# Incident Angle of Saltating Particles in Wind-Blown Sand

**DOI:** 10.1371/journal.pone.0067935

**Published:** 2013-07-09

**Authors:** Lin-Tao Fu, Tian-Li Bo, Hai-Hua Gu, Xiao-Jing Zheng

**Affiliations:** 1 Key Laboratory of Mechanics on Environment and Disaster in Western China, Ministry of Education, Lanzhou University, Lanzhou, China; 2 Department of Mechanics, School of Civil Engineering and Mechanics, Lanzhou University, Lanzhou, China; Plymouth University, United Kingdom

## Abstract

Incident angle of saltating particles plays a very important role in aeolian events. In this paper, the incident angles of sand particles near the sand bed were measured in wind tunnel. It reveals that the incident angles range widely from 0° to 180° and thereby the means of angles are larger than published data. Surprisingly, it is found the proportion that angles of 5°–15° occupy is far below previous reports. The measuring height is probably the most important reason for the measurement differences between this study and previous investigations.

## Introduction

Saltation is the key factor in the wind-blown sand movement [1]. The saltating particles shape the land surface [2] and load the atmosphere with mineral dust [3], which therefore causes the change of landscape and climate on both Earth and Mars [4]. With the development of computers, numerical simulation becomes an important way to study the movement of wind-blown sand, particularly on Mars. However, except for two models [5], [6] in which each collision is simulated through contact potentials in MD-simulation to simulate the sand bed process at particle scale, many models encounter the same problem that it is difficult to determine the information of lift-off particles after sand-bed collision [7].

**Figure 1:**
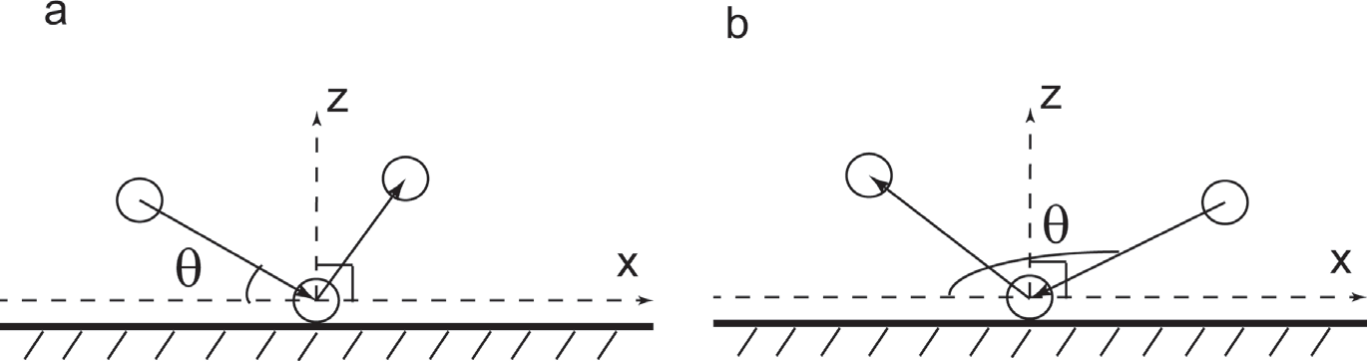
A sketch of incident angle. x and z represent the stream-wise and the vertical direction, respectively. θ is the incident angle. (a) shows the case of θ∈[0°,90°) and (b) tells the case of θ∈[90°,180°].

The information of lift-off particles typically depends on the incident speed and the incident angle of saltating particles. Here, the incident angle is defined as angle between the incident velocity and the horizontal level (See Fig. 1). Both experimental and simulated results [8], [9], [10], [11], [12] suggest that the number of ejected particles is linearly related to incident speed, i.e., *N*≈*A*.*V_imp_*, where *A* is the coefficient and *V_imp_* is the incident speed. This relation is widely employed in numerical models. But, in many models, the coefficient *A* is taken as a constant which includes gravity, particle characteristic diameter and particle mass [13]. Besides, according to wind tunnel experiment [10], the restitution coefficient of incident speed is usually chosen as a value 0.5–0.6. Therefore, it seems that the incident angle of particles is considered as a constant in many past and current studies.

In fact, both the number of ejected particles and the restitution coefficient vary with incident angles. Experimental results [12], [14], [15] indicate that the number of ejected particles increases but the restitution coefficient decreases with the rise of incident angle. The incident angle was taken as a constant because some wind tunnel experiments [10], [16], [17], [18] showed a narrow range (5°–15°) of incident angle. In such a narrow range, the variation of both the number of ejected particles and the restitution coefficient caused by the change of angle is small.

However, the range of incident angles in wind-blown sand may be wider than those classic reports. Jensen and Sorensen [19] reported a wider range of incident angles by considering the saltating particles with low trajectories. The wind tunnel measurements of Dong et al. [20] and Kang et al. [21] reveal that the incident angles range from 0° to180°. The wind tunnel study of Rasmussen and Sorensen [22] shows there are many particles backward flying against the wind near the sand bed, which also suggests the wide range of incident angle. Apart from the difference in distribution range, the distribution laws from experiments are different. The results of Kang et al. [21] show the distribution of incident angles follows a negative exponent law. But the results of Dong et al. [20] reveal a more complex distribution, rather than the negative exponent law. Thus, the treatment, that the incident angle was taken as a constant, may be over-simplified. Therefore, we need some more experiments to study the incident angle of particles in wind-blown sand.

In addition, once the incident angles distribute widely, it becomes important and necessary to investigate the incident angle distribution of each incident speed. Rice et al. [11] studied the relation between lift-off speed of ejected particles and lift-off angle of those particles. It reveals that ejected particles with a small speed have a wide range of lift-off angles as well as a large mean of angles but those particles with a large speed have a narrow range and a small mean of angles. It is interesting to find whether the relation between incident angle and incident speed follows the law between angles of ejected particles and speeds of those particles.

Therefore, this paper is aimed to investigate the incident angle of saltating particles near the sand bed and the influence of incident angles on the evolution of wind-blown sand. The experiment design is introduced in Methods section. In Results and Discussion section, the distributions and the means of measured incident angles in various cases are given and some discussion also is followed. A concise conclusion is arranged in the end.

## Methods

**Figure 2:**
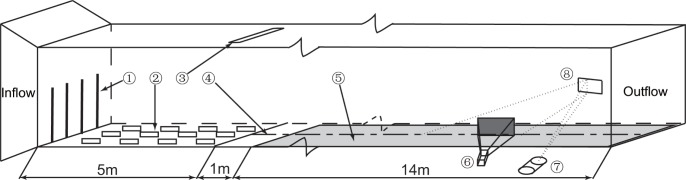
The diagrammatic sketch of wind tunnel experiment design. ① and ② are triangular turbulence spires and solid roughness elements respectively. ③ represents the sand supply system. ④ is the central axis line of wind tunnel. ⑤, ⑥, ⑦ and ⑧ represent the sand bed, CCD camera, laser generator and reflecting mirror respectively.

The experiments were conducted in a multiple environmental wind tunnel located at Lanzhou University. The wind tunnel was described in detail by Tong and Huang [23]. The working section of the tunnel is 20 m in length. The sand material comes from Badain Jaran Desert with the average diameter of 0.3 mm. The thickness of sand bed is 4.5 cm and the length of sand bed is up to 14 m. The experiment design in this paper is followed the work of Rasmussen et al. [24] (Details see Fig. 2). Triangular turbulence spires and solid roughness elements were placed at the front of working section to help the development of boundary layer. A sand supply system was fixed at the end of roughness elements. Thus, the experiments were carried out in a transport limited condition but not a supply limited condition.

**Figure 3:**
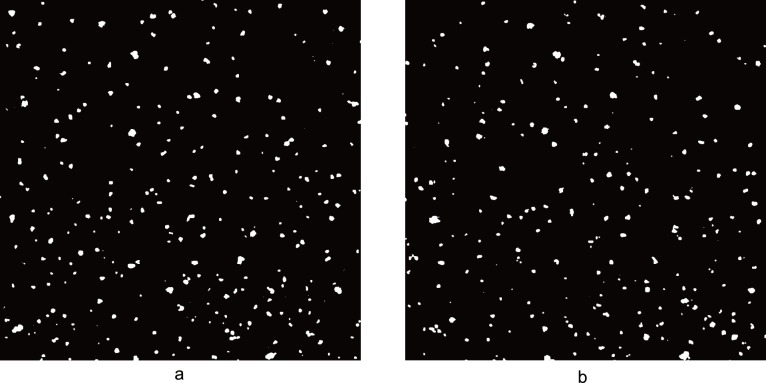
Part of an example image pair. (a) shows the former image in a pair and (b) reveals the latter one. In the image, the white spots represent the particles flying in the air.

The wind profile in the wind-blown sand was measured by the outdoor constant temperature anemometer (Details see Fig. A in File S1). The motion of particles was measured by a PTV system [25], [26]. During the experiments, the CCD camera was arranged at 2 m before the ending point of working section. Unlike many research before, the laser sheet was not casted into the sand bed vertically but approached the bed with an angle. This treatment can decline the influence of light reflection of sand bed on the measuring results. The distance between camera and layer sheet is 65 cm. The camera can capture 7 pairs of pictures per second. Fig. 3 shows a part of an example image pair. The time delay between the two pictures in each pair is 0.2 ms. The camera was set to capture pictures at a resolution of 2048×2048 pixel^2^.

During each run, three steps were followed. First, the sand bed was smoothed to keep the thickness of bed unchanged. Second, prior to measurements, both the wind tunnel and the sand supply system have been running for a short time to ensure the well development of wind-blown sand. This delay of time depends on wind strengths. In case of weak strength the delay is about 30 s, while the delay reduces to about 10 s for strong strength. Finally, the motion of sand particles was measured continuously. The duration of the measurement in a run varies with wind strengths. The duration ranges from 350 s to 90 s corresponding to wind strengths. Thus, under every wind strength condition, the captured images are more than 400 pairs and the obtained total samples in the whole image region are up to 10^7^.

Because of the random fluctuation of sand bed and the complex interactions among particles, it is difficult to judge whether the movement of a particle is saltation or creep at the place very close to the bed (e.g. 0–1 mm). Therefore, the speed and the angle of incident particles at 1–3 mm were taken as the incident information of saltating particles. The middle height 2 mm was taken as the mean height of these particles. Here, based on the measured data, we performed statistics for the distribution and the mean of incident angles at 2 mm in various cases of wind strengths (u_*_ = 0.30, 0.39 and 0.48 m/s). Moreover, we conducted statistics for the distribution and the mean of incident angles at 2, 4, 6, 8 and 10 mm at fixed wind strength (u_*_ = 0.39 m/s) respectively. The mean (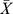) and the corresponding mean square error (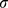) are calculated as 
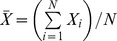
 and 
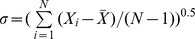
, respectively. Here, *N* is the sample number. In the following section, the results of data statistics and corresponding discussion were shown. The data, used in the following, are uploaded as File S2.

## Results and Discussion

### Wind Profile

Since the size of wind tunnel is large, the thickness of turbulent boundary layer reaches about 40 cm [23]. Vertical profiles of time-averaged streamwise speed *U(z)* ranging from 2 to 32 cm can be described as (Details see Fig. B in File S1): 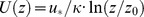. Here, u_*_ is friction speed. z is the vertical coordinate. κ is the von Kármán constant and taken as 0.4. *z_0_* is the aerodynamic roughness length. The roughness could be well fit by the Bagnold roughness law: 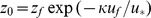, where *z_f_* is the height of the focus point and *u_f_* is the corresponding wind speed (*z_f_* = 6 mm, *u_f_* = 2.7 m/s).

### Population Distribution and Mean Value of Incident Angles

**Figure 4:**
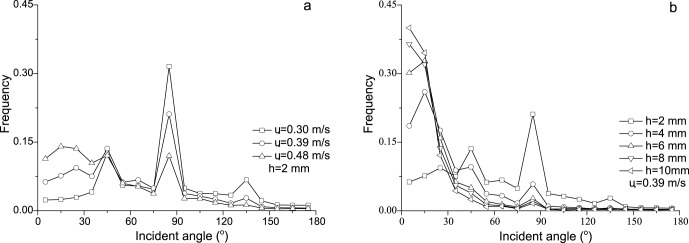
The population distributions of incident angles in different wind strengths (a) and measuring height (b).

**Figure 5:**
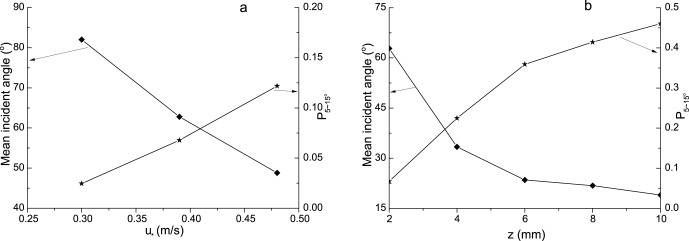
The mean incident angle and the proportion of 5°–15° incident angles (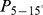) in different wind strengths (a) and measuring height (b). The diamond and the star symbols represent the mean incident angle and 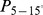, respectively.

From Fig. 4a, it can be seen that the distribution of measured incident angles ranges from 0° to 180°. This wide range is quite different from the results of many previous studies [10], [16], [17], [18] which suggest a narrow distribution of incident angles. But, it is consistent with the measurements of Dong et al. [20] and Kang et al. [21]. The wide distribution of incident angles may be caused corporately by sand-bed collision [5], mid-air collision of sand [27] and turbulence of boundary layer [28]. The results of Kang et al. [21] reveal that the distribution of incident angle follows a negative exponent law in cases of wind strengths. But both our results and the results of Dong et al. [20] reveal a more complex distribution, rather than the negative exponent law. In particular, our results show a large proportion of sand particles have an incident angle near 90°, which is also found in the study of Dong et al. [20]. This is possibly caused by the large proportion of saltating particles with low trajectories in such low height [19]. In addition, the distributions obtained by both us and Dong et al. [20] vary with the change wind strengths. In fig. 5a, the means of incident angles obtained from our data range from 48° to 82°. They are larger than previous studies, including the measurements of Dong et al. [20] and Kang et al. [21]. Furthermore, the statistics for the proportion
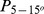
that angles of 5°–15° occupy were performed (Fig. 5a). Results indicate that the proportion 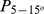 is far below previous thought. Although the proportion rises with the increase of u_*_, it only reaches 12% at the maximum measuring wind strength.

In our opinion, the measuring height is probably the most important reason for the measurement differences between our results and published data. Because of the limitation of technique, scientists [14], [16], [17], [18], [25] usually measured the motion of particles at the place far from the bed (generally higher than 5 mm) where the particle density is low. Thus, they lost the information of particles with lower trajectories [19]. Dong et al. [20] and Kang et al. [21] used the PDA and PDPA to measure the movement of sand particles much closer to the bed respectively and they therefore found the wide distribution of incident angles. Also, at a high place, the speed of a particle is usually dominated by its horizontal speed component, which therefore leads to a small incident angle. The statistics for the distributions of incident angles at wind strength u_*_ = 0.39 m/s along height also were conducted (Fig. 4b). It can be found that the distribution of incident angle trends to be a negative exponent law with the raise of height (Details see Table S1 in File S1). The means of incident angles along height also are calculated (Fig. 5b). It reveals that the means of incident angles vary from 63° to 19° with the increase of height. The proportion that small incident angles occupy increases along with increasing height. In detail, the proportion, that angles of 5°–15° occupy, rises quickly with height and soon reaches up to about 50% (Fig. 5b). Thus, it is reasonable to explain why previous studies showed the means of incident angles varied among 10°–20° and most of incident angles distributed between 5°–15°.

Apart from the measuring height, the finite size of wind tunnel affects the transportation of sand and the effect becomes significant with the decrease of the size [29]. One way to solve the problem is providing a sand supply at the front of sand bed [24]. Although Rice et al. [10], [11] provided a sand supply, the distance between the supply point and the measuring point is not long enough.

### The Angle Distribution and Mean Angle of Incident Particles with the Same Speed

**Figure 6:**
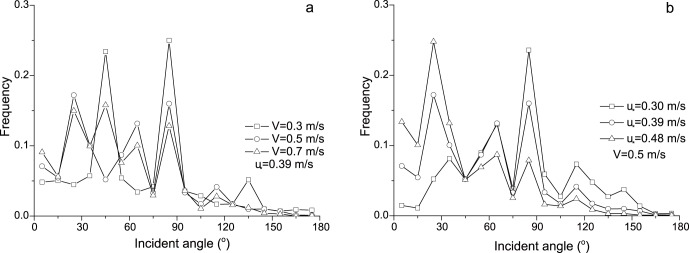
The distributions of incident angles of different particle speeds for a fixed wind strength (a) and of different wind strengths for a fixed particle speed (b).

**Figure 7:**
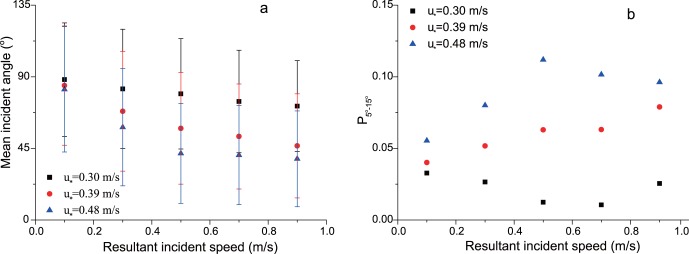
The mean incident angle (a) and the proportion of 5°–15° incident angles (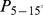) (b) versus resultant incident speeds of particles.

In this section, the angle distributions of incident particles with the same speed at the height of 2 mm were analyzed. Here, the speeds of sand particles in a speed interval are represented by the middle value of the interval. The interval of speed in this paper is 0.2 m/s. That is, for example, if V = 0.5 m/s, it says the speeds of particles range from 0.4 to 0.6 m/s. From Fig. 6a, it can be seen that the angles of particles for the same speed distribute widely from 0° to 180° but most of them are located within 0°–90°. The distributions of incident angle vary with the change of particle speed. For instance, when V = 0.3 m/s, the distribution shows us bimodality; while the distribution shows three peaks when V = 0.5 m/s. These various distribution laws should be caused by the complex interactions between incident particles and sand bed as well as other saltating particles. With the increase of incident speeds, the proportion of small angles increases and that of large angles decreases accordingly. The wind strength also affects the distribution of incident angles. Fig. 6b shows the distributions of incident angles when the incident speed is 0.5 m/s. It suggests that the proportion of small angle increases with wind strengths. Moreover, the means of incident angles in cases of incident speeds were carried out (Fig. 7a). It reveals that the mean value of incident angles decreases with incident speeds. This law is agreed with the study of Rice et al. [11]. The explanation of the variation of incident angle should trace back to the definition. Since the change of vertical component of particle speed in wind-blown sand is small relative to horizontal component, a large incident speed usually means a large stream-wise component and therefore a small incident angle. The change of the proportion 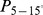 with both incident speeds and wind strengths is shown in Fig. 7b. It can be seen the proportion 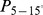 almost doesn’t change with incident speeds when u_*_ = 0.30 m/s. But with the increase of wind strengths, the proportion 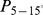 goes up with incident speeds. Therefore, the incident angle could be regarded as the function of incident speeds and wind strengths. In addition, the Fig. 7b also tells that the proportion 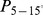 is very low.

Based on the results above, we made some discussion here. The wider distribution of and very low proportion of 5°–15° incident angles make the predominant role of small incident angle in wind-blown sand challengeable. The predominant role of small incident angle has been stated in previous studies because some experiments [30], [31] show only very fast particle can lead to splash. For instance, the works of Beladjine et al. [30] and Oger et al. [31] suggest that the lowest speed of particle with diameter of 0.3 mm for splash is about 2.2 m/s. And, such large speed of particles usually means a small incident angle in wind-blown sand. Thus, the key to this problem is the minimum incident speed for splash.

In fact, there are two works indicating a lower threshold value. The studies of Werner [15] and Anderson and Haff [8] suggest the minimum impact speed for particle with diameter of 0.3 mm is about 1.0 m/s or lower. Furthermore, our experimental data at u_*_ = 0.3 m/s show that there are only several particles with a speed larger than 2.2 m/s, but a few particles with a speed larger than 1.0 m/s. Therefore, it is possible that the works of Beladjine et al. [30] and Oger et al. [31] overestimate the below incident speed, and thus the particle with a relative large incident angle also could lead to splash. The main difference in below incident speed among experiments may come from experimental materials. The material used in Beladjine et al. [30] and Oger et al. [31] is the PVC bead with the diameter of 6 mm. But Werner [15] and Anderson and Haff [8] conducted their experiments by real sand.

Because we lack systematical measurement of minimum incident speed for splash in wind-blow sand, we could not make sure the contribution of small incident angle to the ejection of particles quantitatively. However, we do believe the important role of the particles with small incident angle, because small angle often results in high probability of rebound apart from ejection of particles [12].

### Conclusions

In this paper we measured the incident angles of saltating particles in wind-blown sand by PTV technique. It reveals that the incident angles near the sand bed range widely from 0° to 180°. The wide distribution of incident angles may be caused corporately by sand-bed collision, mid-air collision of sand and turbulence of boundary layer. The proportion that angles of 5°–15° occupy is far below previous reports. The measuring height is probably the most important reason for the measurement differences between our results and published data. The measured results along with the height provide us the reasons why previous studies showed the means of incident angle varied among 10°–20° and most of incident angles distributed between 5°–15°. The incident angle could be regarded as the function of incident speeds and wind strengths. Although the proportion of 5°–15° incident angles is low, however, the importance of small incident angles is undoubted. We could not make sure the contribution of small incident angle to the ejection of particles quantitatively. More systematical measurements are expected to determine the role of small incident angle in wind-blown sand. Besides, since the incident angles are positively related to the release of mineral dust [32] and the abrasion of land surface [33], the study of this work may be helpful to improve the understanding of these aspects in aeolian research.

## Supporting Information

File S1 **This file contains Figure A, Figure B and Table S1.** Figure A shows the set of anemometer in the wind tunnel. Figure B says the vertical profiles of time-averaged stream-wise velocity. Table S1 tells the fitting parameters of the distributions of incident angles.
(DOC)

File S2 **This file contains experimental data of seven measured conditions.** These data files are named as “S-experimental data”. The S-experimental data 1–1 is measured with friction wind speed u_*_ = 0.30 m/s and the measuring height z = 2 mm. The S-experimental data 2-1, 2-2, 2-3, 2-4 and 2-5 are measured at z = 2, 4, 6, 8 and 10 mm, respectively, with the same u_*_ = 0.39 m/s. The S-experimental data 3-1 is measured with u_*_ = 0.48 m/s and z = 2 mm.
(RAR)
