# Correction: Incident Angle of Saltating Particles in Wind-Blown Sand

**DOI:** 10.1371/annotation/8a3d1140-8ca3-4f8e-a4a0-8f47f6622056

**Published:** 2013-09-19

**Authors:** Lin-Tao Fu, Tian-Li Bo, Hai-Hua Gu, Xiao-Jing Zheng

Multiple funding organizations and grants were incorrectly omitted from the Funding Statement. The Funding Statement should read: “This research was supported by grants from the National Natural Science Foundation of China (Numbers 11072097, 11232006, 11202088, 10972164, 11121202), National Key Technology R&D Program (2013BAC07B01), the Science Foundation of Ministry of Education of China (Number 308022), Fundamental Research Funds for the Central Universities (Numbers lzujbky-2009-k01, lzujbky-2012-203) and the Project of the Ministry of Science and Technology of China (Number 2009CB421304). The authors express their sincere appreciation to the supports. The funders had no role in study design, data collection and analysis, decision to publish, or preparation of the manuscript.” 

