# Supplementary material for: Incident Angle of Saltating Particles in Wind-Blown Sand
Source: PLoS One. 2013 Jul 9;8(7):e67935. doi: 10.1371/journal.pone.0067935 (PMC3706613; doi:10.1371/journal.pone.0067935)
Supplement: File S1 — This file contains Figure A, Figure B and Table S1. Figure A shows the set of anemometer in the wind tunnel. Figure B says the vertical profiles of time-averaged stream-wise velocity. Table S1 tells the fitting parameters of the distributions of incident angles. (DOC) [file pone.0067935.s001.doc]

**Supporting Information**

A. Anemometer in wind tunnel experiment

Figure A. The set of anemometer in the wind tunnel.

The wind profile in the wind-blown sand was measured by the outdoor constant temperature anemometer (made by DANTEC). They are located at 3m before the ending point of working section. The frequency of measurements is 2Hz. Measuring heights of the design are 2, 4, 8, 16 and 32cm respectively.

B. Wind profile

Figure B. Vertical profiles of time-averaged streamwise velocity

From the figure S2, we can see that the vertical profile of time-averaged streamwise velocity in the well-developed wind-blown sand could be described as: $U\left(z\right)=u_{*}/\kappa\cdot ln\left(z/z_{0}\right)$. Here, *u** is friction velocity. κ is the von Kármán constant and taken as 0.4. *z_0_* is the aerodynamic roughness length. The three estimated wind strengths are 0.30, 0.39 and 0.48 m/s and the corresponding roughness are 0.15, 0.39 and 0.59mm respectively. The roughness could be well fit by the Bagnold roughness law: $z_{0}=z_{f}\exp(-\kappa u_{f}/u_{*})$, where *z_f_* is the height of the focus point and *u_f_* is the corresponding wind velocity(*z_f_*=6mm，*u_f_*=2.7m/s).

C. The distribution law of incident angles along with height

The following table 1 lists the curve-fit parameters for the distributions of incident angles in cases of measuring heights at wind strength u*=0.39m/s. Here, the distributions are assumed as a negative exponent law$p(\theta)=A\exp(-B\theta)$. The least square method is employed and the correlation coefficients (R^2^) are shown.

Table S1. Fitting parameters of the distributions of incident angles

| Height (mm) | A | B | R^2^ |
| --- | --- | --- | --- |
| 2 | 0.11 | 0.008 | 0.23 |
| 4 | 0.28 | 0.026 | 0.86 |
| 6 | 0.43 | 0.041 | 0.91 |
| 8 | 0.51 | 0.050 | 0.95 |
| 10 | 0.57 | 0.055 | 0.94 |
